# Paper-Based Colorimetric Biosensor for Tear Glucose Measurements

**DOI:** 10.3390/mi8040104

**Published:** 2017-03-29

**Authors:** Ellen Flávia Moreira Gabriel, Paulo Tarso Garcia, Flavio Marques Lopes, Wendell Karlos Tomazelli Coltro

**Affiliations:** 1 Instituto de Química, Universidade Federal de Goiás, 74690-900 Goiânia, GO, Brazil; ellenflavia.moreira@hotmail.com (E.F.M.G.); ptarsogarcia@gmail.com (P.T.G.); 2 Faculdade de Farmácia, Universidade Federal de Goiás, 74605-170 Goiânia, GO, Brazil; flaviomarques.ufg@gmail.com; 3 Instituto Nacional de Ciência e Tecnologia em Bioanalítica (INCTBio), 13083-970 Campinas, SP, Brazil

**Keywords:** bioanalytical chemistry, clinical diagnostics, glucose enzymatic reaction, non-invasive diagnostics, paper microfluidics, point-of-care testing

## Abstract

This paper describes a paper-based colorimetric biosensor for measuring glucose concentration levels in human tear samples. Colorimetric biosensors were wax printed on paper platforms and modified with chitosan previously prepared in acetic acid. The proposed device was explored to measure the glucose levels in human tear samples using 3,3′,5,5′-tetramethylbenzydine (TMB) as the chromogenic reagent. The paper-based colorimetric biosensor exhibited a linear behavior for the glucose concentration range between 0.1 and 1.0 mM. The achieved analytical sensitivity and limit of detection (LOD) were 84 AU/mM and 50 µM, respectively. Moreover, the device provided analytical reliability and no statistical difference when compared to the data recorded with a commercial glucometer. The proof-of-concept of our device was successfully demonstrated by measuring the glucose levels in six tear samples from nondiabetic subjects. In general, the results showed that the colorimetric biosensor has noticeable potential to be used as a powerful tool for tear glucose monitoring, since this fluid offers lower potential interferences, non-invasive sample collection and is pain-free. Furthermore, the proposed device could facilitate the treatment of diabetic patients who need constant control of glucose levels and cannot tolerate multiple finger sticks per day.

## 1. Introduction

For many years, glucose has been one of the most globally important biological compounds clinically investigated. The broad interest in this analyte is related to diabetes, a metabolic and chronic disease that causes abnormal levels of glucose in the blood [1,2,3,4]. According to the World Health Organization (WHO), the number of people that live with diabetes almost quadrupled in the last 24 years. It is estimated that ca. 8.5% of the world’s population is diagnosed with diabetes [5]. The main problem of diabetes is related to other health complications that it can cause. Diabetes can lead to loss of vision, cardiovascular disease and kidney problems. Furthermore, it damages the capillary blood vessels, causing non-healing wounds that can lead to limb amputations [2].

The most common practice to monitor glucose levels is via self-monitoring using blood as the biological fluid. Nowadays, different commercial systems are available for purchase where a small blood drop can be obtained from the fingertip or forearm using a lancet [6]. As disadvantages of diabetes personal control are the pain and invasiveness caused to patient that need use this system several times during a day. In consideration of reducing the discomfort to patients with diabetes, many efforts have been dedicated to the development of personal glucose sensors with pain-free and non-invasive methods [2,4,6,7]. Recently, Mitsubayashi et al. reported a review on the development of cavitas sensors for measuring glucose using breath, sweat, saliva and tears as samples [3]. The latter appears as a promising biological sample for non-invasive diagnostics due to its easy accessibility and evidential correlation with blood glucose concentration levels [8]. In general, tear fluid is composed of more than 20 components that include minerals, proteins, glucose and metal ions, and most of these components are present in low concentrations [2,4]. The glucose concentration levels, for example, range from 0.1 to 0.6 mM [2,9]. In this way, many research groups have analyzed tears using fluorescence (LIF) [10,11], mass spectrometry (MS) [12,13], electrochemical [1,2,6,7,14,15] and colorimetric [16,17,18] detectors. These detection methods have exhibited suitable sensitivity and high selectivity for tear glucose analysis. In addition, the required sample volume in each mentioned system is considerably low(ca. 10 µL) [2]. All these features are advantageous for exploiting tear samples for non-invasive diagnostics while still offering great potential for point-of-care testing (POCT).

One of the first reports describing the use of a non-invasive colorimetric biosensor for glucose analysis in human tears was published in 1987 by Romano et al. [16]. The authors demonstrated a colorimetric reaction with triphenyl-tetrazolium chloride on filter paper strips imbibed with tears. A good correlation between blood and tear glucose levels was observed for samples collected from patients in the first stage of diabetes. Later, Kim and co-workers [17] developed a microfluidic paper-based analytical device (µPAD) for quantitative determination of glucose in tears based on colorimetric detection. For this purpose, the authors used a mixture of diethyl phenylenediamine and 1-chloro-4-naphtho as a chromogenic agent and observed a linear behavior for the glucose concentration range between 1 and 10 mg/dL. The glucose levels achieved in tear samples exhibited a good relationship with the values found in blood. More recently, Gabriel et al. [18] showed that the modification of the paper surface with chitosan promotes noticeable improvements in the sensitivity and detectability levels. The improvement in terms of color intensity is associated with the formation of chitosan films on cellulose fibers, which creates a suitable environment for the fast electron transfer between the enzyme and sensing surface. The use of a chromogenic solution composed of 4-aminoantipyrine (4-AAP) and 3,5-dichloro-2-hydroxybenzenesulfonic acid (DHBS) (4 mM:8 mM) provided a limit of detection (LOD) value of 23 µM, one of the lowest LODs reported for glucose using µPADs [18]. The enhanced analytical performance allowed the detection of glucose in human tears.

The µPADs have emerged as a disposable platform for POCT due to their attractive advantages including low cost, biocompatibility, global affordability and minimal sample/reagent consumption. Recently, this microfluidic platform has been explored in different applications including environmental monitoring [19,20,21], food and water analysis [22,23,24], forensic studies [25,26] and clinical diagnostics [27,28,29,30,31,32,33,34]. Most of the applications on µPADs are performed in association with colorimetric detection, which is one of the simplest and cheapest detectors for use in POCT [27]. The increasing popularity of colorimetric detection on µPADs is associated with the possibility of using cell phone cameras, scanners, digital cameras or portable microscopes for image acquisition [35,36,37]. These electronic devices are considerably inexpensive, portable and globally affordable. Despite the advantages offered by colorimetric measurements, the poor homogeneity of the developed color inside the detection zones is one of the major problems that may compromise the chemical analysis. However, recent advances showing the modified paper surface [18,38,39,40,41] have enhanced the analytical performance of µPADs and provided accuracy and reliability for clinical assays.

In this current report, the main goal was to develop a simple wax-printed device for measuring glucose levels in tear samples using colorimetric detection. Initially, the paper surface was modified with chitosan to better support the attachment of enzymes on its surface, as recently reported [18]. Differently from the previous report, the µPAD configuration was designed in a simple format (24 mm × 10 mm) containing a sample inlet zone, a control zone and a detection zone. A comparison between the data recorded on paper-based devices and the values obtained using a commercially available personal glucometer was also presented to evaluate the analytical reliability of the proposed device. Lastly, the clinical feasibility was demonstrated in the analysis of glucose in tear samples from six nondiabetic patients.

## 2. Materials and Methods

### 2.1. Chemicals and Materials

Glucose oxidase (from *Aspergillus niger*, 181 U mg^−1^) (GOx), horseradish peroxidase (73 U mg^−1^) (HRP), 3,3′,5,5′-tetramethylbenzydine (TMB), methanol, acetic acid, glucose, chitosan powder from crab shells (degree of deacetylation > 75.0%), sodium phosphate monobasic anhydrous and sodium phosphate dibasic anhydrous were purchased from Sigma-Aldrich (St. Louis, MO, USA). All chemicals were used as received and prepared in ultrapure water (18 MΩ cm^−1^, Millipore, Kansas City, MO, USA) without further purification. Chromatography paper (CHR) grade 1 was purchased from Whatman (Maidstone, Kent, UK). Scanner (model Scanjet G4050) was acquired from Hewlett-Packard (Palo Alto, CA, USA).

### 2.2. Fabrication of µPADs

**Figure 1:**
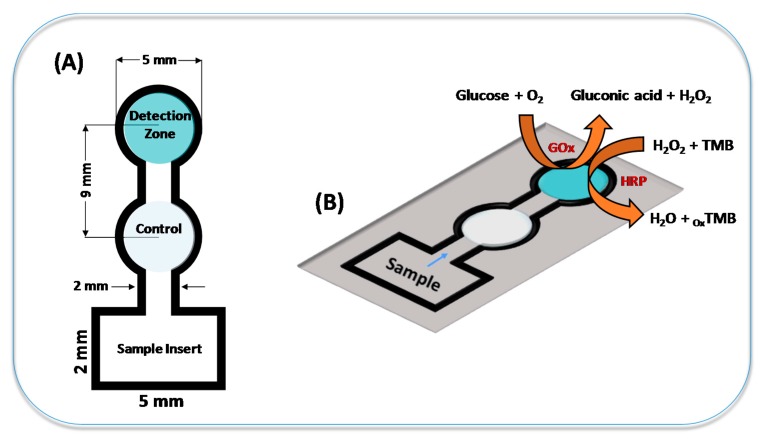
Presentation of (**A**) the layout of µPAD used for glucose colorimetric assays and (**B**) a simplified view of the enzymatic reaction involved in the presence of chromogenic reagent (TMB) for glucose detection.

Paper microfluidic devices were produced by wax printing [42]. Briefly, the desired geometry was designed on Corel Draw^TM^ graphical software (Version X3) and printed on paper substrates using a wax printer (Xerox ColorQube 8570, Xerox Corporation, Rochester, NY, USA). Afterwards, effective hydrophobic barriers were created by melting the printed wax at 180 °C for 120 s using a hot plate. Finally, one side of paper devices was covered with adhesive tape to prevent sample leaking. As showed in Figure 1A, the µPAD layout contains two circular zones (diameter of 5 mm each), named as a control and detection zones, and a square region dedicated to sample inlet. The control zone was used to detect potential interferent compounds and minimize the matrix effect. The circular zones and the square region were interconnected by a microfluidic channel defined with 14 mm length and 2 mm width. The dimensions of the finished μPADs were 24 mm × 10 mm.

### 2.3. Modification, Enzymatic Reaction and Colorimetric Detection

Prior to enzymatic reaction and colorimetric detection, µPADs were initially modified with chitosan according to a procedure recently reported by our group [18]. First, a chitosan solution was prepared in 2% (*v*/*v*) acid acetic. Then, ca. 2 µL of this solution was added into the control and detection zones and allowed to dry at room temperature for 15 min. Afterwards, detection zone was spotted with a chromogenic solution composed of TMB (15 mM) followed by adding of an enzymatic mixture containing GOx (120 U mL^−1^) and HRP (30 U mL^−1^) prepared in 100 mM phosphate buffer solution (pH = 6.0). The control zone was spotted only with the enzymatic solution. Lastly, sample aliquots (5 µL) were introduced in the sample inlet zone and allowed to reach the detection zones under lateral flow. Colorimetric detection was performed with an office scanner (Hewlett-Packard, model Scanjet G4050) using 600-dpi resolution. All images were scanned 15 min after sample addition. The recorded images were converted to Red-Green-Blue (RGB) scale and analyzed in Corel Photo-Paint^TM^ software (Version X3).

### 2.4. Sample Collection

Six human tear samples were donated by a local Ophthalmological Institute (Goiânia, GO, Brazil). These samples were collected from six patients through borosilicate glass microcapillary tubes (Microcaps^®^, Drummond Scientific Company, Broomall, PA, USA). All samples were used freshly without pretreatment and dilution step.

## 3. Results and Discussion

### 3.1. Optimization Process and Analytical Performance

The chemical modification of µPADs with chitosan has ensured high sensitivity and, consequently, low detectability levels for the colorimetric analysis of glucose based on enzymatic assays, as recently reported [18]. For this reason, this strategy was adopted to investigate the analytical performance of µPADs as a portable and reliable platform for tear glucose measurements. Firstly, the required volumes of chitosan, reagent and the sample were optimized. The highest color intensity was achieved using 2.0 µL of chitosan solution, 1.5 µL of reagent and 5.0 µL of sample.

**Figure 2:**
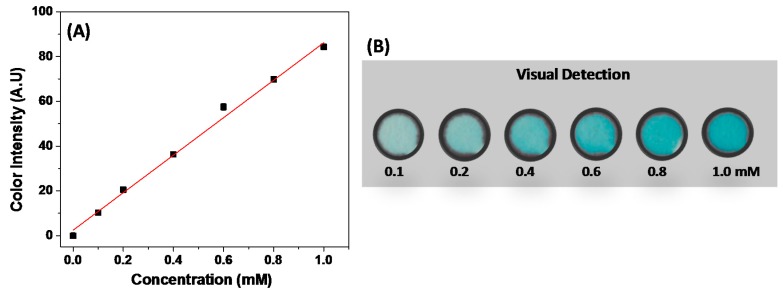
(**A**) Analytical curve for glucose assay using TMB as the colorimetric indicator. The regression linear equation obtained was *y* = 2.462 + 83.728*x* with a *R*^2^ of 0.995; (**B**) Color scale for visual detection of tear glucose levels.

The proposed device offered excellent reproducibility. The calculated relative standard deviation (RSD) value over 10 measurements in different devices was ca. 2%. The analytical performance of the μPAD treated with chitosan was investigated using the optimized conditions. For this purpose, an analytical curve for glucose was obtained in the concentration range from 0 to 1 mM, as can be seen in Figure 2A. The found analytical sensitivity and LOD values were 84 AU/mM and 50 µM, respectively. The LOD values were calculated based on the ratio between three times the standard deviation of the blank and the angular coefficient of the analytical curve. The linear concentration range and the analytical sensitivity achieved using TMB as a chromogenic agent were similar to the parameters recorded using a mixture composed of 4-AAP and the DHBS colorimetric indicator [18]. Although the LOD achieved using TMB was higher to the value reported with 4-AAP/DHBS [18], it is suitable for glucose analysis in tear samples, where the glucose concentration levels range commonly between 0.1 and 0.6 mM. In this case, the choice of indicator did not promote noticeable differences in the analytical performance of wax-printed μPADs.

Considering the use of similar instrumentation for image acquisition, the LOD described in this study is similar to that found by Figueredo et al. [41] and lower than other values achieved on μPADs previously oxidized with sodium periodate [39], modified with silica nanoparticles [40] or even without any modification [35,43,44]. On the other hand, the performance of chitosan-modified µPADs is superior to that recently reported by other groups. Chun and co-workers reported a paper-based glucose biosensing system utilizing a smartphone as a colorimetric detector [37]. The authors found a LOD of 0.3 mM and successfully detected glucose in serum samples. Oyola-Reynoso and co-workers developed a µPAD based on the drawing of hydrophobic barriers with a ball-point pen [45]. Although the LOD has not been reported, the analysis of glucose revealed poor color uniformity and significant background coloration. Taking into account the analytical performance, the use of chitosan has promoted high sensitivity and low detectability levels in comparison with other reports found in the literature, as mentioned above.

The use of cell phone cameras appears to be a current trend in the development of portable instrumentation [36,37]. However, some drawbacks associated with the camera resolution and quality, the distance of image capture and the control of ambient light still require systematic investigation to make these systems capable of reliable chemical analysis in POCT. To make the detection of glucose as simple as possible and instrument-free, a color scale was created based on the relationship between the glucose concentration and color intensity. As can be seen in Figure 2B, the gradual increase of the color intensity corresponds to the higher concentration of glucose. The visual colorimetric detection can be helpful to allow rapid access to the glucose concentration levels, thus contributing mainly to diagnosis or monitoring at the point-of-care.

### 3.2. Analytical Reliability

**Figure 3:**
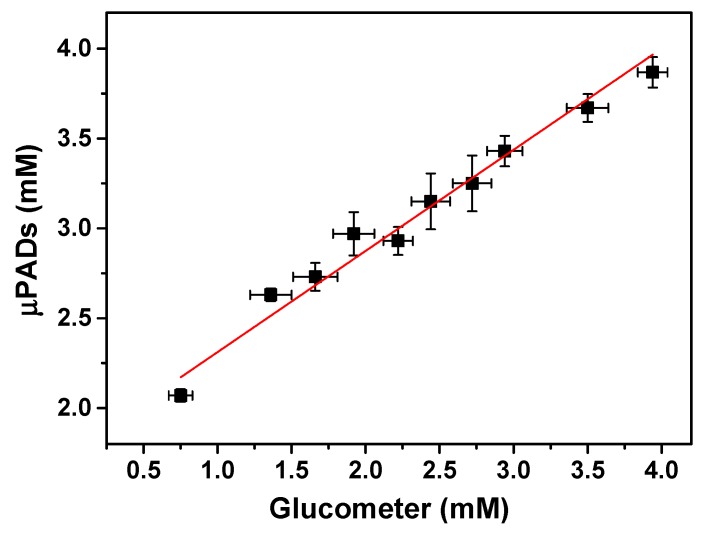
Regression curve showing the comparison between the proposed µPADs and commercial glucometer for glucose analysis. The curve was obtained through the analysis of 10 standard solutions of glucose prepared at different concentrations.

The analytical reliability of the colorimetric measurements for the glucose assay was compared to the signal recorded in a commercial glucometer (Accu-Check@ Active, Roche Diagnostics, Mannheim, Germany). The comparison between both techniques was realized using the regression curve [46]. For this purpose, 10 standard solutions containing glucose were prepared at different concentrations and analyzed through both the µPADs and glucometer. The colorimetric response on the µPADs was recorded as described in the Experimental Section. On the other hand, the analysis on the glucometer was carried out by the addition of a small drop of each solution on a disposable test strip. The glucose level appears on the meter display in the mg dL^−1^ unit. The values found with both techniques were converted to the mM scale and the correlation curve is shown in Figure 3.

The equation obtained from the curve presented in Figure 3 was *y* = (1.82 ± 7.66) + (0.53 ± 3.04)*x*. Confidence limits for the intercept and slope were calculated and the results show that the confidence intervals include the values 0 and 1 for the intercept and slope, respectively. Since an ideal situation requires a 0 value for the intercept and a 1 value for the slope, it can be inferred that the proposed method has no significant difference from the commercial test at a confidence level of 95% (*p* = 0.05).

### 3.3. Tear Glucose Analysis

As previously mentioned, human tears have been widely exploited in recent years as a fluid for clinical analysis [3,4]. The broader interest in this biological fluid is due to the direct correlation of the analyte concentration with other body fluids such as serum and blood [8,12]. Furthermore, the sample collection process is a non-invasive and pain-free method. Besides, tear samples can be analyzed without previous pretreatment, which enable their determination at the point-of-care using a portable and disposable device. In order to monitor glucose in tear samples, many flexible sensors have been presented in the literature [3]. In general, these sensors are fabricated by depositing electrochemical trials over the contact lens and placed directly on the eye [8,9]. In this case, the sensors continuously monitor the glucose levels using a minimal volume of tears.

**Figure 4:**
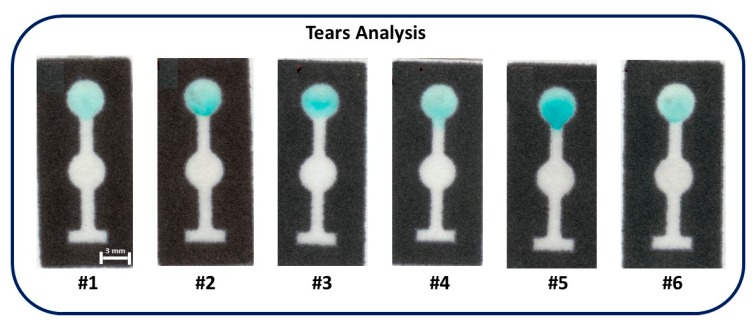
Optical micrographs showing colorimetric measurements of glucose in six tear samples donated from nondiabetic volunteers.

Paper-based devices modified with chitosan appear as simple and alternative sensors to monitor glucose levels in tear samples without any damage to health. The glucose concentrations were determined in samples from six nondiabetic patients. Samples were directly analyzed using TMB as the indicator. Figure 4 shows the optical micrographs for glucose analysis in six human tear samples. The glucose concentration levels found were 120 ± 10 µM (sample #1), 280 ± 10 µM (sample #2), 240 ± 10 µM (sample #3), 105 ± 10 µM (sample #4), 340 ± 10 µM (sample #5) and 110 ± 10 µM (sample #6). These values are in agreement with the normal levels of tear glucose reported in the literature [2,6,9,12].

## 4. Conclusions

In summary, a paper-based colorimetric biosensor previously modified with chitosan was developed for tear glucose monitoring at point-of-care. The proposed devices offer great sensitivity and detectability to proceed with real sample analysis. The analytical response based on the color intensity was directly compared with a personal glucometer and no statistical difference was found at a confidence level of 95%. The paper-based biosensor was used to analyze six samples donated by nondiabetic volunteers. The concentration range was found between 105 ± 10 and 340 ± 10 µM. Based on the data found in the literature, these values are in agreement with concentration levels normally present in tear samples. Lastly, the presented data have demonstrated the clinical feasibility of the proposed µPADs, as well as their potential for use as low-cost, pain-free and non-invasive biosensors in glucose monitoring.
